# Desired Characteristics of a Clinical Decision Support System for Early Sepsis Recognition: Interview Study Among Hospital-Based Clinicians

**DOI:** 10.2196/36976

**Published:** 2022-10-21

**Authors:** Jasmine A Silvestri, Tyler E Kmiec, Nicholas S Bishop, Susan H Regli, Gary E Weissman

**Affiliations:** 1 Palliative and Advanced Illness Research Center University of Pennsylvania Perelman School of Medicine Philadelphia, PA United States; 2 University of Pennsylvania Health System Philadelphia, PA United States; 3 Division of Pulmonary, Allergy and Critical Care Medicine University of Pennsylvania Perelman School of Medicine Philadelphia, PA United States; 4 Leonard Davis Institute of Health Economics University of Pennsylvania Perelman School of Medicine Philadelphia, PA United States; 5 Penn Institute for Biomedical Informatics University of Pennsylvania Perelman School of Medicine Philadelphia, PA United States

**Keywords:** sepsis, predictive information, clinical decision support, human factors, sepsis onset

## Abstract

**Background:**

Sepsis is a major burden for health care systems in the United States, with over 750,000 cases annually and a total cost of approximately US $20 billion. The hallmark of sepsis treatment is early and appropriate initiation of antibiotic therapy. Although sepsis clinical decision support (CDS) systems can provide clinicians with early predictions of suspected sepsis or imminent clinical decline, such systems have not reliably demonstrated improvements in clinical outcomes or care processes. Growing evidence suggests that the challenges of integrating sepsis CDS systems into clinical workflows, gaining the trust of clinicians, and making sepsis CDS systems clinically relevant at the bedside are all obstacles to successful deployment. However, there are significant knowledge gaps regarding the achievement of these implementation and deployment goals.

**Objective:**

We aimed to identify perceptions of predictive information in sepsis CDS systems based on clinicians’ past experiences, explore clinicians’ perceptions of a hypothetical sepsis CDS system, and identify the characteristics of a CDS system that would be helpful in promoting timely recognition and management of suspected sepsis in a multidisciplinary, team-based clinical setting.

**Methods:**

We conducted semistructured interviews with practicing bedside nurses, advanced practice providers, and physicians at a large academic medical center between September 2020 and March 2021. We used modified human factor methods (contextual interview and cognitive walkthrough performed over video calls because of the COVID-19 pandemic) and conducted a thematic analysis using an abductive approach for coding to identify important patterns and concepts in the interview transcripts.

**Results:**

We interviewed 6 bedside nurses and 9 clinicians responsible for ordering antibiotics (advanced practice providers or physicians) who had a median of 4 (IQR 4-6.5) years of experience working in an inpatient setting. We then synthesized critical content from the thematic analysis of the data into four domains: clinician perceptions of prediction models and alerts; previous experiences of clinician encounters with predictive information and risk scores; desired characteristics of a CDS system build, including predictions, supporting information, and delivery methods for a potential alert; and the clinical relevance and potential utility of a CDS system. These 4 domains were strongly linked to clinicians’ perceptions of the likelihood of adoption and the impact on clinical workflows when diagnosing and managing patients with suspected sepsis. Ultimately, clinicians desired a trusted and actionable CDS system to improve sepsis care.

**Conclusions:**

Building a trusted and actionable sepsis CDS alert is paramount to achieving acceptability and use among clinicians. These findings can inform the development, implementation, and deployment strategies for CDS systems that support the early detection and treatment of sepsis. This study also highlights several key opportunities when eliciting clinician input before the development and deployment of prediction models.

## Introduction

### Background

Sepsis, a life-threatening dysregulation of the immune system in response to an infection, is a significant risk for patients and a major burden for health care systems in the United States, with over 750,000 cases annually and total costs nearing US $20 billion [1,2]. The hallmark of effective sepsis treatment is early recognition and initiation of broad-spectrum antibiotic therapy [3,4]. However, sepsis is characterized by high diagnostic and prognostic uncertainty, which often results in delayed recognition and treatment, especially among patients who develop sepsis in hospitals.

To facilitate the timely recognition and management of sepsis, several machine learning prediction algorithms have been developed and integrated into electronic health record (EHR)–based alerts and clinical decision support (CDS) systems [5-7]. Although deployment of such CDS systems is common, there is little high-quality evidence to suggest that they are reliably effective in improving care processes or clinical outcomes [5,6]. Several prior studies have identified barriers to successful integration of sepsis CDS systems into clinical practice, including poor diagnostic accuracy, poor education and implementation strategies, and clinician mistrust of an unfamiliar system [8,9]. At the same time, human factors research into sepsis-specific CDS systems has focused on the display of visual information but has overlooked team-level dynamics and clinician-level affective and cognitive influences [10-12]. User interfaces for predictive models, often built with complex statistical learning algorithms, may present clinicians with outputs that are difficult to explain and that sometimes contrast with clinical intuition, thereby decreasing the likelihood of adoption [12,13].

### Objective

To address these research gaps, we sought to elicit perspectives on and preferences for a hypothetical, sepsis-focused predictive CDS system from a multidisciplinary group of hospital-based clinicians who regularly care for patients suspected of sepsis. We used qualitative semistructured interviews informed by human factors methodology to identify important clinician perspectives on the clinical and team-level context for using an alert. Our goal was to elicit this information to inform the design and implementation of a future early-warning sepsis CDS tool.

## Methods

### Overview

To investigate how a sepsis CDS system might integrate into clinical workflows and to elicit clinicians’ perspectives on and preferences for prediction information, we used human factors methodologies of contextual inquiry and cognitive walkthrough (we met over video rather than physically in the participants’ workspaces as a necessary modification because of constraints of the COVID-19 pandemic).

### Vignette and Simulated Chart Development

We first engaged clinicians in diagnosis and decision-making through clinical vignettes in which sepsis was on the differential diagnosis. The study team prepared 2 vignettes (Multimedia Appendix 1) for each patient with possible sepsis varying in severity. Clinical decision-making regarding sepsis treatment is imbued with uncertainty related to both the diagnosis and the severity of the presentation [14]. To explore how a sepsis CDS system might optimally support such decision-making across the full range of diagnostic and prognostic uncertainty, we varied this uncertainty in 2 distinct vignettes. The first vignette was created as a straightforward case that met all sepsis criteria and had no obvious competing diagnosis. The second vignette was created to have higher diagnostic uncertainty, in which the patient met fewer formal criteria and had a broader differential diagnosis of potentially causative disease processes but presented with a higher severity of illness. Our team worked with an EPIC (Epic Systems Corporation) build specialist to develop 2 simulated patient charts in the EHR that reflected the clinical courses described in the vignettes. In addition to reviewing the written vignette, each participant in the first round of interviews was asked to review the simulated data in the EHR and to verbalize their thoughts and considerations in a simulated clinical evaluation. After the research team conducted a preliminary analysis, a list of factors pertaining to sepsis was developed and presented to round 2 participants for review in tandem with the vignettes. This 2-step process allowed the study team to member check our findings from previous interviewees and ensure that we captured a comprehensive list of the relevant factors assessed, completing a differential diagnosis for patients with possible or suspected sepsis.

### Study Population and Recruitment

From September 2020 to February 2021, we recruited a convenience sample of 5 physicians, 4 advanced practice providers (APPs) (nurse practitioners and physician assistants), and 6 bedside nurses who cared for inpatients at the University of Pennsylvania Health System. During round 2 of interviews, we determined that we had reached saturation after 3 participants did not contribute any additional factors to our comprehensive list of data points considered during a hypothetical differential diagnosis of vignette patients. We identified eligible participants through department leaders and staff lists, and sent email invitations to participate in the study. We used a 2-step hierarchical recruitment approach. First, we used purposive sampling to identify a range of specialty wards in the University of Pennsylvania Health System. Second, we recruited clinicians directly via email from these wards using convenience sampling. We chose this method to facilitate the inclusion of a range of perspectives from clinicians in different care settings while also completing recruitment in a timely manner. All participants verbally acknowledged their consent to participate at the onset of the web-based interview and received a US $50 gift card for completing the study.

### Ethical Considerations

This protocol was deemed exempt by the institutional review board of the University of Pennsylvania (protocol number 843819).

### Interview Guide, Data Collection, and Analysis

Interviews were conducted via videoconference by either a qualitative research specialist (JS) or a doctorate-level coinvestigator (SR) with extensive qualitative training. During the interviews, responses were noted by the clinical research coordinators of the research team (TK and NB). To reduce bias regarding the diagnosis of hypothetical patients, no clinically trained research staff or experts in sepsis were present during the interviews. As nurses and physicians discussed the desired aspects of sepsis alerts or other topics of interest, interviewers earnestly followed those lines of inquiry, asking open-ended questions to elicit comprehensive responses. For each interview, participants were presented with both vignettes (a straightforward vignette followed by a more complex and diagnostically ambiguous vignette) and asked questions about their communication and decision-making processes. A subsequent round of EHR walkthroughs and review of pertinent factors when considering sepsis followed. After mentally engaging each participant in this task, we asked questions about their prior experiences with and preferences for predictive information relevant to caring for patients suspected of sepsis. Each interview lasted approximately 45 minutes, was recorded and professionally transcribed, and was deidentified before analysis. This study reports the findings related to preferences for and perspectives on prediction information, while the results focused on decision-making will be reported separately.

We first interviewed 3 clinicians (1 physician and 2 bedside nurses) who were experienced in sepsis management to test our interview guide and simulated EHR data for accuracy and effectiveness. The interview guide was updated iteratively to reflect the emerging themes and questions. All subsequent interviews took place with clinicians who staffed ward units (general medicine, oncology, pulmonary, neurosurgery, and gerontology). The initial interview questions were designed to engage participants in active decision-making and to elicit a differential diagnosis. Subsequent questions sought to elicit the perceived potential impacts of a predictive sepsis alert on workflow and decision-making when diagnosing and managing a patient suspected of sepsis, including preferences for and acceptability of a potential future sepsis alert.

In this qualitative study, we used an abductive analytic approach in which the existing theory can be built upon a combination of inductive and deductive approaches to coding and emphasizing new or surprising findings [15,16]. To inform the development of our codebook, 3 members of the team (JS, SR, and GW) first independently identified themes and then met to discuss commonalities. Additionally, our interview guide was developed to include inquiry into several factors identified in previous research as integral to the development of CDS [17]. Half of the transcripts were reviewed and coded by at least two members of the research team (JS, SR, and GW); all disagreements were reconciled through consensus. The remaining transcripts were coded by a single member of the research team (JS).

## Results

### Overview

We conducted 15 interviews with 5 physicians, 4 APPs, and 6 nurses (Table 1). Through thematic analysis, we identified 4 broad themes linked to the likelihood of adoption and their impact on clinical workflows when diagnosing and managing patients suspected of sepsis. The first theme was clinician perceptions of prediction models, including both positive and negative sentiments that shaped how clinicians viewed predictive information. The second theme was previous experiences of clinician encounters with predictive information and risk scores, both in the context of the local health system and with nationally recognized tools for sepsis identification. The third theme centered on the desired characteristics of a CDS system build and included predictions, supporting information, and delivery methods for a potential alert. The fourth theme included the clinical relevance and potential utility of a CDS system for its intended audience. These themes, including codes, definitions, and examples of each major theme, are detailed in Table 2. In addition, select illustrative quotes are provided to provide additional context to the identified themes.

**Table 1:** Clinical cohort characteristics (N=15).

Participant characteristic		Value
**Age (years), mean (SD)**		
	18-24	1 (6.7)
	25-34	13 (86.7)
	35-44	1 (6.7)
**Sex, n (%)**		
	Male	4 (27)
	Female	11 (73)
**Race, n (%)**		
	Asian or Asian American	2 (13)
	White	11 (73)
	Multiracial	2 (13)
**Hospital role, n (%)**		
	Registered nurse	6 (40)
	Critical care fellow	1 (7)
	Hospitalist	4 (27)
	Nurse practitioner	2 (13)
	Physician assistant	2 (13)
Years in current role, median (IQR)		3.5 (1.5-5)
Years of inpatient experience, median (IQR)		4 (3.5-7)

**Table 2:** Themes derived from interviews with clinicians about their preferences for a sepsis-focused predictive clinical decision support system with definitions and examples.

Theme and definition				Examples
**Clinician perceptions**				
	**Positive sentiments**			
		Statements made that reflect positive feelings or opinions about predictive information. Includes statements that describe building or already having trust in predictive information.	Helpful when the data are not giving a clear picture or unsure of course of action When a prediction is tied to a specific intervention or relevant clinical decision-making Clinician education efforts to explain relevant studies, model validation, and predicted outcomes	
	**Negative sentiments**			
		Statements made that reflect negative feelings or opinions about predictive information. Includes statements that describe losing trust or having mistrust in predictive information.	A clinician feeling like they want to go based off other their own gestalt rather than trusting an alert without a clear explanation Frequently dismissing false positive alerts	
**Previous experiences**				
	**Previously deployed sepsis alerts**			
		Discussion of EHR^a^-based sepsis-specific alerts that were previously or are implemented in the health system.	Two prior iterations of a sepsis-specific EWS^b^ (EWS 1.0 and 2.0)	
	**Risk scores and predictions**			
		Discussion of bedside clinical risk scores that clinicians have experience using. This includes predictive information for both sepsis and other clinical conditions.	Wells’ criteria SIRS^c^ Quick sequential organ failure assessment Ranson score CHA_2_DS_2_-VASc	
**Desired characteristics**				
	**Supporting information**			
		Clinical information contained in a potential alert to illustrate the reasons an outcome may occur. Additionally, any resources that would be available or linked within an alert	Vital sign trends Quantitative presentation of risk information Links to antibiotic decision tree or antibiotic stewardship info to guide treatment decisions	
	**Platform delivery**			
		The interface, vector for delivery, timing of delivery, and placement of a potential alert.	Text alerts BPA^d^	
	**Predictions**			
		Clinical outcomes that may occur in patients who are at risk or have developed sepsis and that would be helpful to predict at the bedside.	Mortality Transfer to intensive care unit Development of sepsis or septic shock	
**Potential utility**				
	**Audience**			
		Discussion of the best recipients to target for receiving a potential alert to render it useful rather than being dismissed.	More useful for novice practitioners Less useful for nurses who do not put in orders Clinicians changing services, infrequently rotating on a service	
	**Clinical impact**			
		The potential impacts of an alert on the course of clinical care.	Change decisions about if and when to initiate broad-spectrum antibiotics Clarifying to users how might clinical care change based on an alert?	

^a^EHR: electronic health record.

^b^EWS: early-warning system.

^c^SIRS: systemic inflammatory response system.

^d^BPA: best practice alert.

### Clinician Perceptions

Clinicians’ perceptions of predictive information, including positive and negative sentiments, are closely linked to their trust in an alert. Many of the study participants shared that a clinician’s knowledge of the background and development of a CDS system contributes to trust in that system when deployed in a clinical setting:

I’m someone that attends grand rounds and evidence-based medicine presentations, so I would be a participant in something like that. And so, that would be a useful way to get the information out. Any information that helps to determine how it was made, I think, whether it’s from studies done at the hospital, or from evidence taken—reviewed from different articles. I think things like that really do carry a lot of weight, especially if there’s something in an algorithm that doesn’t immediately intuitively make—isn’t what you thought it would be. It’s helpful to have information to understand how you got to that point, because then you learn something.

Advanced practice provider 3

Participants described additional methods for clinician engagement and education that would foster acceptance of a new alert, such as presenting information about the alert’s background during pre-existing information sharing venues, distributing previous research, and clinical leadership providing educational opportunities for those using the alert on the floor:

Anytime something new is started on the hospital we always have huddles...sometimes teams will come and say we have this new product that we’re implementing or there’s a new protocol for sepsis, so teams will kind of round...And then also just working with the leadership team because they can—they really disseminate, every week a lot of the leadership teams will disseminate like new things. So the CNS [Clinical Nurse Specialist] and CPL [Clinical Practice Leader] teams do a great job of kind of educating the nurses. So I feel like if there was to be something newly implemented, those teams specifically will help you make a plan on what is possible.

Bedside nurse 3

Clinicians’ assessments may not align with the predictions made on the alert, thereby diminishing trust in the algorithm. Clinicians viewed alerts negatively and felt that they may be inaccurate or fire too frequently:

So, I would like to see something that doesn’t trigger every single time there’s a small heart rate change because maybe my patient just went for a walk with physical therapy or is getting out of bed and their heart rate is 120, but they’re also getting out of bed for the first time in two weeks.

Bedside nurse 4

In addition, alerts were viewed as unhelpful when they were not actionable, with a clear next step toward patient care:

Because, honestly, sometimes there are things in EPIC right now, obviously, that pop up. And they ask you if you would consider sepsis in this patient. But to my knowledge, right now it just shows you a bunch of vital signs and, if you’ve been following the patient for a couple days and you’re pretty confident in your treatment plan, you just kind of hit, okay, no thanks, or no suspected sepsis, and just kind of move on. I mean, I can’t tell you how many times I’ve probably just went ahead and hit that button just to get it off the screen because I’m trying to do something else.

Advanced practice provider 1

### Previous Experiences

Previous experiences with predictive information in the form of clinical risk scores and alerts embedded in the EHR were common among the participants. Clinicians had varied experiences with such risk predictions, which included sepsis-specific tools such as systemic inflammatory response syndrome, quick sequential organ failure assessment, and EHR-embedded predictive alerts. Some clinicians have highlighted risk scores based on their usefulness or lack thereof in making clinical decisions:

I do think it comes down to the whole question of, like, modified SOFA versus qSOFA criteria or even SIRS in the sense that if the patient shows more than like three or four or even five things it’s not going to be very valuable to me if I’m the one who’s responsible for recognizing it in the absence of an alert coming up. And that’s why no one uses the SOFA criteria is because there’s like nine different things and I can’t remember them.

Physician 4

Several clinicians had personal interactions with previously deployed early-warning systems and drew on these experiences to reflect on the usefulness of sepsis-specific CDS systems:

I don’t know if this is still available and in EPIC, and just not at Penn anymore, but I know there used to be a sepsis—a screener tool based on the data that used to pop up. I don’t know if you’re familiar with that. I remember entering certain vital signs and getting a notification that this patient is at risk for sepsis. But I think that was helpful in identifying trends early and that are so slight that nursing probably wouldn’t think anything of. I think they did away with it, at one point, just because of how frequently it was going off and it wasn’t always 100 percent accurate.

Bedside nurse 2

Overall, these alerts were described as unfavorable because of the perceived high frequency and low accuracy, in addition to disrupting the usual clinical workflow.

### Desired Characteristics

Clinicians described the desired characteristics of a potential predictive model and alert, including its predictions of clinical outcomes, supporting information regarding the patient’s status, and the platform on which it is delivered:

Like immunosuppressed status, if they were on immunosuppressive medications, if they have underlying malignancy, if they have risk factors for infection, age, if they are community dwellers or if they’re coming from nursing homes or care facilities, if they’re hospitalized–already hospitalized patients. Those are some of the kinds of things I would be thinking about.

Advanced practice provider 2

Participants sought an alert that would deliver a prediction about a specific clinical outcome in the near future. Risk of mortality, future requirement for intensive care unit transfer, need for antibiotics or mechanical ventilation, and being at risk for sepsis or septic shock were suggested as helpful clinical outcomes to present in an alert. Clinicians also expressed a preference for numeric data to contextualize a patient’s risk for a specific outcome. Although there was no agreement regarding the specific thresholds, many clinicians felt that plain language such as “your patient has an 80% chance of developing septic shock in the next 24 hours” would be clinically actionable:

If someone told me this patient, based—has a likelihood of, I don’t know, greater than 30 percent in-hospital mortality I might be more likely to pull the trigger on antibiotics....Other criteria – I guess you could say chance of discharge to home versus a rehab facility. In my mind, that shows whether we caught the infection quick enough so that their level of debilitation was less and they can go home or if they were so debilitated because we waited so long that they now need to go to a physical therapy skilled nursing facility for a couple of weeks. Other things, I think percent chance that they have to go to the ICU, for example, because maybe right now when I recognize the infection and sepsis you don’t need to go to the ICU, but if someone told me this patient has a 33 percent chance of going to the ICU, I might be inclined to act quicker.

Physician 3

In addition, it is important for clinicians to present appropriate supporting information regarding a patient’s clinical presentation to contextualize risk prediction. Trends such as for fever, heart rate, and laboratory values were of particular importance to clinicians who prioritized tracking a patient’s trends over time rather than viewing an isolated value. Additional desired alert features included trends in laboratory results and vital signs, reduction of false alarms, and explanatory content about what variables drive a model’s prediction:

Like again, to incorporate the idea of a trend. Like how new is this abnormality, and abnormality meaning combining not just vital signs, but lab values and orientation status and all of these things that come with sepsis.

Bedside nurse 4

I guess, to me, the trends are so helpful that I know where to find them. But it’s not super intuitive, in Epic when you see a white count, you kind of need to scroll to see what the white count has been.

Advanced practice provider 4

There was a consensus among participants that unobtrusive alert delivery methods would be the best. Clinicians favored an easy-access location such as the patient summary tab or chart advisories section in the EHR or a flag highlighting the value of concern when viewing laboratory results. Alerts that presented as a hard stop (such as a best practice advisory) by requiring acknowledgment before navigating other areas of the electronic medical record were viewed unfavorably. Alerts with *soft* stops—notifications not requiring user acknowledgment—that provide guidance for future action and could be accessed at the clinicians’ convenience, were preferred:

Just stick it in with the chart advisories. It pops up every once in a while. Not every single time you open EPIC, but the first time after it generates the chart advisory, you have to acknowledge it, give a reason like provider notified. And then it goes away for a while. It doesn’t keep coming up every time you open the goddamn chart.

Bedside nurse 5

### Potential Utility

Clinicians felt strongly that an alert’s utility would be integral to its success. We identified the delivery of relevant information to the appropriate audience and positive impacts on clinician care as 2 important factors contributing to the perceived utility of a CDS system. Participants from all clinical roles suggested directing alerts to specific units where sepsis is not as common or to clinicians who do not rotate frequently in wards where sepsis is seen:

For those of us who see–as internal medicine people who treat infections all the time, I don’t think it’s like that helpful unless you’re pretty novice. Where I think this is most helpful is to the person who does not usually take care of these types of patients, so like to some degree surgery or folks who are in ultra-subspecialties that would always defer this to someone.

Advanced practice provider 2

Some nurses felt that an alert would be more beneficial for the clinician responsible for placing orders than for the entire team, as the clinician is less likely to observe incremental temporal changes in the patient yet is charged with making antibiotic administration decisions:

So, what I’m saying is it would be more helpful for providers who are not with the patient at bedside, and who sometimes never actually even see the patient with their own eyes. So, especially on night shift. On night shift, the provider does not come see the patients unless there’s some clinical indication that the nurse has brought to their attention.

Bedside nurse 5

There was agreement among clinicians regarding the need for an actionable alert that directly affects patient care, such as more frequent monitoring, ordering additional or repeat testing, and initiation of a sepsis protocol or antibiotics:

A big thing is, of course, appropriate use of antibiotics to make sure that we’re not over treating patients. And so, maybe something that reminds you to reevaluate your antibiotic use at the 24-hour mark is something that could be helpful. Because a lot of times when we’re not sure what’s going on, we do add a lot on initially, and then we get a lot more information and then we –it is appropriate to start peeling things back. Other times it’s not, and someone –and we don’t find out what’s going on, and so we continue to treat someone empirically. But something like that could be helpful to prompt you to really think about the antibiotic decisions that you’re making and to think about antimicrobial stewardship.

Advanced practice provider 3

In addition, physicians and APPs recognized the potential of alerts to have positive effects on antibiotic stewardship, while nurses noted being able to use a previous CDS tool to facilitate advocating for patients and prompting conversations with the larger care team:

When this alert would go off, yes, you had to notify the charge nurse and the team member had to come to the bedside right away and you had to like make a plan and say like, okay, we’re going to draw cultures. Now I feel like – we don’t have an alert, but the nurses and the whole team does a really good job of like alerting the team, making sure it’s like a phone call and advocating for blood cultures and all of those things now...So I feel like we’ve had a really good – since they stopped that alert, I haven’t seen – I’m unaware of like the nursing not notifying the team and advocating for the right things or the team not starting things appropriately.

Bedside nurse 3

## Discussion

### Principal Findings

In preparation for building and implementing a predictive sepsis CDS alert in an academic health system, we interviewed physicians, APPs, and nurses about their experiences and perceptions of predictive CDS systems in clinical workflows around patients suspected of sepsis. We identified themes in these interviews that offer insights into strategies to increase the likelihood of adoption, increase clinical effectiveness, and establish trust in an alert among hospital-based clinicians. These findings have several implications for developing sepsis-focused decision support tools and providing guidance for the creation of trusted and actionable CDS systems.

### Clinician Perceptions

Participants expressed interest in clinician engagement and educational activities regarding a predictive CDS system before deployment. Specifically, participants expressed an interest in education on how the model was developed, what specific factors went into the predictions, and how to interact with a predictive alert. These findings complement previous work, suggesting that training and education on the growing presence of artificial intelligence in health care could extend to the organizational level to increase machine learning literacy in clinical staff and overcome some of the barriers in CDS adoption [9,18]. Interviewed clinicians who were either previously exposed to or educated on CDS model development had a much more favorable perception of the system. Opportunities for education and interaction between clinicians and the CDS development team should be a part of any new CDS system integration Previous evidence shows a lack of coordinated implementation strategies lowers the likelihood of adoption of sepsis predictive alerts in multiple previous CDS explorations [19].

These findings highlight the benefits of a prospective assessment strategy, rather than a retrospective one, because the impact of a CDS is greatly influenced by stakeholder adoption and buy-in [20,21]. This is in contrast to recent qualitative work in this field that has focused primarily on clinician perceptions of existing and previously implemented sepsis alerts [8,9]. Although still useful, these retrospective analyses are limited to the characteristics of alerts that were already developed and implemented.

### Previous Experiences

Clinicians’ frustrations were evident in “hard-stop” alerts that require user action or acknowledgment, commonly citing experiences with best practice advisories that were viewed as unhelpful and contributing to workflow delays. The effects of such undesirable CDS characteristics can lead to alert fatigue and interruptions in cognitive and clinical workflows, which in turn leads to delays in the initiation of antibiotics [22-24]. A total of 2 previous mixed methods studies evaluating clinician perceptions of a previously deployed sepsis early warning system in our health system reported low clinical relevance and low likelihood of affecting clinical patient management [8,17]. These alerts, especially those contradicting a clinician’s impression of a patient, were met with a lukewarm response from clinicians, as teams were required to meet and discuss the patient within a short period, similarly interfering with the normal clinical workflow on the ward [17].

### Desired Characteristics

Importantly, we identified preferences for alert characteristics that are unique to both bedside nurses and clinicians responsible for ordering antibiotics. Participants in the nursing group spoke about how sepsis-specific predictive CDS systems might support the need both to advocate for patients and to relay critical information to other clinicians who make decisions on ordering antibiotics and other diagnostic tests. Preferences expressed by bedside nurses extend and complement the findings of previous studies that showed nursing preferences for alerts that provide timely care recommendations, highlight treatment protocols, and address a patient’s condition, rather than those that emphasize regulatory guidelines [25]. Although bedside nurses are not consistently included as recipients of sepsis-based electronic alerts [26,27], these findings underscore their importance in caring for patients suspected of sepsis and how a sepsis CDS system might address some of the challenges that they face in relation to information gathering and team communication.

Physicians and APPs identified specific elements of a patient’s EHR data and history that could help them in their treatment decisions. A patient’s past antibiotic history, microbiological data, and comorbidities, such as underlying malignancies and immunosuppressed status, were all recognized by multiple clinicians as especially relevant in determining appropriate antibiotics to be used during treatment initiation. Clinicians also expressed a preference for specific antibiotic guidance to balance therapeutic efficacy and stewardship. A sepsis-focused CDS should provide easy access to these EHR data elements for clinicians to facilitate decision-making based on alerts. Notably, these treatment-focused data elements were not highlighted in the nurses’ responses, which were, in contrast, more focused on immediate patient care concerns and communicating risks to the clinicians responsible for placing orders. Although some of our findings, such as the desire to see data trends in the CDS system, are consistent with previously reported results [17], our results extend prior work in this area by identifying additional features of both the alert itself and the health system’s approach to engaging clinicians before deploying an alert that can inform the planning of future development and deployment of sepsis-specific CDS systems.

### Potential Utility

Our participants desired an easy-to-digest alert that was, most importantly, accessible, unobtrusive, and believed to be clinically accurate. The acceptability of sepsis CDS relies on both its prediction accuracy and its presentation of information in a readily interpretable manner [28]. There is a growing body of evidence investigating the importance of human factors in CDS design, ranging from alert type to textual and graphical displays of information [25,28]. Clinicians made numerous suggestions along these lines, including displaying a “flag” or marker in the “summary” tab of a patient’s chart to assist the clinician in recognizing an issue without interrupting usual workflows. Nurses specifically highlighted the advantage of even a small visual signal to review the patient’s trending information, allowing them to take a closer look at patient temporal data in cases that they otherwise may not have.

A sepsis CDS system represents a complex interaction between technological factors such as flagged alerts and display of information, and social factors such as communication between nurses and physicians. In designing and implementing such a system, detailed aspects of both technology and its use in the hospital setting should be considered. Sociotechnical theory takes a measured approach to these interactions and, over the years, multiple frameworks have been developed to provide a conceptual structure in system design. The Systems Engineering Initiative for Patient Safety model [29] emphasizes the interaction between people, technology, environment, and organization [30]. Another example, Sitting and Singh’s health care sociotechnical framework [31,32], contains 8 dimensions detailing computing infrastructure, the human-computer interface, clinical content, and organizational policies that are used to assess barriers and facilitators when implementing systems. The health care sociotechnical framework is particularly applicable in sepsis CDS design. Granular technology details can be described in the framework, such as the distinction between a *soft stop* and *hard-stop* alert as discussed by our clinicians and the specific steps one would take to acknowledge such an alert. Considering these distinctions early in system design will increase the end user acceptance and utility of a sepsis CDS. The social dimensions of the framework describe the interactions between the clinical staff *users* and those who design, develop, and implement these systems. Explorations such as the one we conducted are the first step in the successful development of a sepsis CDS that considers user perceptions, past experiences, and desired alert characteristics with a high likelihood of clinical utility.

### Limitations

Our study had several limitations. First, all participants had <10 years of experience in an inpatient setting. Thus, this study does not reflect the preferences of more experienced clinicians with distinct practice patterns or different experiences with predictive CDS systems. Second, we only interviewed clinicians from a single health system, and the findings may not be generalized to other health systems with different patient populations, cultures, EHR systems, and previous experiences with sepsis-focused predictive CDS systems. However, illustration rather than generalizability is the intended goal of qualitative research, and the approach outlined here provides a framework for eliciting clinician preferences locally and prospectively, which can be adapted elsewhere.

### Conclusions

This study provides a more detailed understanding of clinician preferences for predictive alerts to assist in the care of patients with a potential sepsis diagnosis. Physicians, APPs, and bedside nurses desire a sepsis-focused predictive sepsis CDS system that is trusted, unobtrusive, and viewed as actionable at the bedside. Opportunities exist for sepsis CDS systems not only to improve diagnosis and treatment decisions but also to facilitate communication in a multidisciplinary team setting. Eliciting stakeholder feedback and identifying preferences for predictive alerts before model development offers an opportunity to engage in clinician education and outreach, which may improve the acceptability and adoption of future sepsis CDS systems.

## Appendix

Multimedia Appendix 1 Nursing and physician interview guides used during semistructured interviews.

## Abbreviations

APP: advanced practice provider
CDS: clinical decision support
EHR: electronic health record
