# Supplementary material for: Desired Characteristics of a Clinical Decision Support System for Early Sepsis Recognition: Interview Study Among Hospital-Based Clinicians
Source: JMIR Hum Factors. 2022 Oct 21;9(4):e36976. doi: 10.2196/36976 (PMC9636532; doi:10.2196/36976)
Supplement: Multimedia Appendix 1 [file humanfactors_v9i4e36976_app1.docx]

**Multimedia Appendix 1.** Nursing and physician interview guides used during semistructured interviews.

***NURSING INTERVIEW GUIDE***

***Intro****:* Thank you for participating in this focus group. Our goals today are:

1. To better understand your role on a care team when decisions are being made to determine if a patient is developing (or has developed) sepsis and when to treat a patient for sepsis by prescribing antibiotics,
2. What information is most important to you when contributing to decision-making, and what other information might you wish you could have, and
3. In what context(s) would the presentation of additional information be most useful.

***PART 1 – Vignettes***

First, we ask you to listen to this scenario (*read one vignette at a time and conduct discussion after each one. For round 1 interviews, the interviewer will open up PennChart and “drive” through the HER as clinicians call out where they would look for information to inform their differential diagnosis. For round 2 interviews, the note taker will be marking off line items in the excel as we go through the open-ended questions*)

***Vignette #1***

You are starting your shift on Tuesday morning as the nurse on a medical ward. You get nursing handoff report from the night team and hear the story of Ms. Jones, who you will be caring for today. The night team reports that Ms. Jones is a 70-year-old woman with history of mild COPD, hypertension, well-controlled diabetes, now admitted to the hospital overnight with fevers, abdominal pain, a high white blood cell count, and a strong suspicion of a urinary tract infection, for which she is being treated with a narrow-spectrum antibiotic. The night team reports that Ms. Jones was more confused overnight, sometimes more somnolent and speaking nonsense sentences, but that the family said she gets confused sometimes so they didn’t make too much of it.

This morning, you go to evaluate Ms. Jones after the shift report and notice that she is arousable after some moderate stimulation, her blood pressure is 100/60 mm Hg, her heart rate is 120 beats per minute in an irregularly irregular rhythm, and her oral temperature is 101.4 F.

***Vignette #2***

You are starting your shift on Thursday night as the nurse on a medical ward. You get nursing handoff report from the day team about a patient, Mr. Smith, who you will be caring for tonight. He was admitted one week ago for a newly diagnosed pulmonary embolism and a maculopapular rash all over his body. He is being treated with therapeutic anticoagulation and has been hemodynamically stable during his admission. Every night he has had low-grade fevers around midnight but has felt comfortable, infectious workups to date have been negative, and he has not been initiated on antibiotics. Each morning the team has also noted a rapidly rising eosinophil count of unknown etiology.

Following the shift report, you go to the bedside to evaluate Mr. Smith. He is awake, interactive, and comfortable appearing, but you notice he is mildly diaphoretic, his heart rate is newly 120 beats per minute in a regular, narrow-complex rhythm, his oral temperature is 101.6 F, and his blood pressure is 120/65 mm Hg.

***Round 1 Vignette Questions***

1. Would you suspect possible sepsis at this point?
2. If you did suspect sepsis, what would your next course of action be?
   - Please direct us to any information you would check on in PennChart to aid in your decision making.
   - Speak aloud as you are reviewing the information to let us know what you are looking for and thinking about as you navigate the patient’s chart.
   - If there is information you wish you had, call that out as well.
   - What questions or interactions, if any, would you ask of other member of the patient’s care team to aid in your decision making?

***Round 2 Vignette Questions***

1. Would you suspect possible sepsis at this point? Describe what leads you to that conclusion.
2. If you did suspect sepsis, what would your next course of action be?
   - Interactions with the care team
   - Consulting the EMR
3. If you determined that the patient had or was developing sepsis, **what would you change in the patient’s care, including monitoring, orders, or other aspects of care?**

***Checklist***: During our first round of interviews we developed a list of data points that providers would consult when making determinations about sepsis. I’ll screen share the list for you to review. Can you call out anything you would or wouldn’t do.

***PART 2 - Predictive Information***

Let’s discuss now what assistance might be valuable from a predictive algorithm.

1. **What has your experience has been, if any, of using predictions or risk scores in clinical practice?**
   - **What made or would make you trust them more or less?**
   - **Did you change clinical practice based on the new information? Why or why not?**
2. We are considering producing a predictive algorithm that could assist in decision making about if, when, and which antibiotics to prescribe for sepsis**. What information, if any, would you find beneficial and actionable from such an algorithm?**
   - *(optional based on interview*) You mentioned <*summarize what additional information they said they wished they could have*> as information you wish you had to assist in your decision-making. **Are there any other types of information you wish you could have?**
3. When would you want to see it so that it could best assist you in deciding a course of action
   - are there specific contexts in the EHR where you would like to see the alert information.
   - Examples:
     - Summarize temporal trends, vital signs, temp, some other info, or measure of variability
     - Prediction – what would you want to see to understand why the prediction was made

***PHYSICAIN AND APP INTERVIEW GUIDE***

***Intro:*** Thank you for participating in this interview. Our goals today are:

1. To better understand the workflow and decision-making process you use in deciding if a patient is developing (or has developed) sepsis, and when to treat a patient for sepsis by prescribing antibiotics,
2. What interactions you have with other members of the patient’s care team to inform your decision, and
3. What information is most important to you when making your decisions, and what other information you might wish you could have.

***PART 1 – Vignettes***

First, we ask you to listen to this scenario (*read one vignette at a time and conduct discussion after each one. For round 1 interviews, the interviewer will open up PennChart and “drive” through the HER as clinicians call out where they would look for information to inform their differential diagnosis. For round 2 interviews, the note taker will be marking off line items in the excel as we go through the open-ended questions)*

***Vignette #1***

You are starting your shift on Tuesday morning on the hospitalist service on a general medical ward. You get signout from the night team and hear the story of Ms. Jones, who you will be caring for today. The night team reports that Ms. Jones is a 70-year-old woman with history of mild COPD, hypertension, well-controlled diabetes, now admitted to the hospital overnight with fevers, abdominal pain, a high white blood cell count, and a strong suspicion of a urinary tract infection, for which she is being treated with a narrow-spectrum antibiotic. The night team reports that Ms. Jones was more confused overnight, sometimes more somnolent and speaking nonsense sentences, but that the family said she gets confused sometimes so they didn’t make too much of it.

This morning, you go to evaluate Ms. Jones after signout and notice that she is arousable after some moderate stimulation, her blood pressure is 100/60 mm Hg, her heart rate is 120 beats per minute in an irregularly irregular rhythm, and her oral temperature is 101.4 F.

***Vignette #2***

You are starting your shift on Thursday night on the hospitalist service on a general medical ward. You get signout from the day team about a patient, Mr. Smith, who you will be caring for tonight. He was admitted one week ago for a newly diagnosed pulmonary embolism and a maculopapular rash all over his body. He is being treated with therapeutic anticoagulation and has been hemodynamically stable during his admission. Every night he has had low-grade fevers around midnight but has felt comfortable, infectious workups to date have been negative, and he has not been initiated on antibiotics. Each morning the team has also noted a rapidly rising eosinophil count of unknown etiology.

Following the day team’s report, you go to the bedside to evaluate Mr. Smith. He is awake, interactive, and comfortable appearing, but you notice he is mildly diaphoretic, his heart rate is newly 120 beats per minute in a regular, narrow-complex rhythm, his oral temperature is 101.6 F, and his blood pressure is 120/65 mm Hg.

***Round 1 Vignette Questions***

1. Would you suspect possible sepsis at this point?
2. If you did suspect sepsis, what would your next course of action be?
   - Please direct us to any information you would check on in PennChart to aid in your decision making.
   - Speak aloud as you are reviewing the information to let us know what you are looking for and thinking about as you navigate the patient’s chart.
   - If there is information you wish you had, call that out as well.
   - What questions or interactions, if any, would you ask of other member of the patient’s care team to aid in your decision making?

***Round 2 Vignette Questions***

1. Would you suspect possible sepsis at this point? Describe what leads you to that conclusion.
2. If you did suspect sepsis, what would your next course of action be?
   - Interactions with the care team
   - Consulting the EMR
3. If you determined that the patient had or was developing sepsis, **what would you change in the patient’s care, including monitoring, orders, or other aspects of care?**

***Checklist***: During our first round of interviews we developed a list of data points that providers would consult when making determinations about sepsis. I’ll screen share the list for you to review. Can you call out anything you would or wouldn’t do.

***PART 2 - Predictive Information***

Predictive algorithms draw on large amounts of data over time to make predictions about what is likely to happen during a current encounter. Let’s discuss now what assistance might be valuable from a predictive algorithm.

1. **What has your experience has been, if any, of using predictions or risk scores in clinical practice?**
   - **What made or would make you trust them more or less?**
   - **Did you change clinical practice based on the new information? Why or why not?**
2. We are considering producing a predictive algorithm that could assist in decision making about if, when, and which antibiotics to prescribe for sepsis**. What information, if any, would you find beneficial and actionable from such an algorithm?**
   - *(optional based on interview*) You mentioned <*summarize what additional information they said they wished they could have*> as information you wish you had to assist in your decision-making. **Are there any other types of information you wish you could have?**
3. When would you want to see it so that it could best assist you in deciding a course of action
   - are there specific contexts in the EHR where you would like to see the alert information.
   - Examples:
     - Summarize temporal trends, vital signs, temp, some other info, or measure of variability
     - Prediction – what would you want to see to understand why the prediction was made
